# A new open dataset from a milling process – data for classification and estimation of tool life

**DOI:** 10.1038/s41597-025-04923-y

**Published:** 2025-04-17

**Authors:** Grzegorz Piecuch, Tomasz Żabiński

**Affiliations:** Department of Computer and Control Engineering, Faculty of Electrical and Computer Engineering, Rzeszow University of Technology, Rzeszow, Poland

**Keywords:** Mechanical engineering, Electrical and electronic engineering

## Abstract

This data descriptor introduces a data set from a CNC machining process which includes vibration and current data recorded for 14 cutting tools used from their initial state until failure. Cuboidal samples made of 42CrMo4 material were used and milled clockwise. The research setup (Haas VF-1 and Beckhoff PAC system), experimental procedures and data were described. The data set has been made publicly available for further research and development and contains all raw and aggregated data with metadata from 968 milling cycles. Data can be used for the classification of tool condition or fault prediction, which is widely used in intelligent prediction maintenance systems.

## Background & Summary

The 4th Industrial Revolution, which emphasizes automation, robotization, and digitalization of industrial processes, is currently underway. Currently, efforts are gradually being made to gradually implement the principles of Industry 5.0. However, many industrial facilities still do not meet the standards of Industry 4.0. One such standard is the self-diagnosis of machinery, commonly referred to as Predictive Maintenance (PdM). In numerous cases, systems are still unable to detect and notify operators of imminent failures or tool damage, which would reduce unexpected downtimes and disruptions to the production chain.

For instance, within machining processes, extensive research efforts are being made to develop effective PdM systems. Most experiments are carried out on materials such as inconel (widely used in the aerospace industry)^1–3^, aluminium^4–7^, and steel^5,8–10^. Measurement data are commonly collected using vibration^11–14^, acoustic emission^15,16^, and force sensors^15,17,18^, including those mounted on spindles and work tables^19–21^. In many studies, metrics such as RMS, crest factor, kurtosis, and skewness^22–24^ are calculated in the time domain from the gathered data. In addition, the time-frequency domain is often used, particularly for vibration signal analysis.

Many studies are unique, using proprietary datasets from real processes; however, a significant number are based on shared datasets^25–27^, to which our dataset is also attached. In this article, we present a new unique database, containing data from the entire life cycle of 14 milling tools that operate under various conditions. A total of 968 milling cycles were recorded. Raw and aggregated data are available. They contain measurements of fast-changing signals (vibration acceleration) and slow-changing signals (current intensity). Metadata are also available, allowing one to link technological data to specific files with measurements. Based on the dataset, algorithmic work can be performed related to both tool condition classification and regression, estimating the time to tool damage. We hope that the new database will have a significant influence on the development of new diagnostic methods.

The presented dataset was used in the other authors work described in^28^ and^29^. The first introduces an innovative approach to identifying and segmenting milling phases. This method involves pre-processing data and analysing time-series signals to detect specific patterns or features that indicate each phase of the milling process. When applied to smart structures or digital twins, this segmentation method provides information comparable to tomography imaging, hence it is referred to as virtual tomography. This phase segmentation method is crucial for the industrial implementation of technological process measurement and diagnostic systems.

The second article^29^ explores the identification of an appropriate regression model to estimate the lifespan of cutting tools in the milling process by analysing the R² parameters of these models. During the study, support vector regression (SVR), decision trees, and neural networks were evaluated. The findings indicate that the highest prediction accuracy with the lowest error values was achieved using two-dimensional neural networks with the LBFGS solver (93.9%) and similarly, SVR also yielded high accuracy (93.4%).

## Methods

### Experimental testbed

**Fig. 1:**
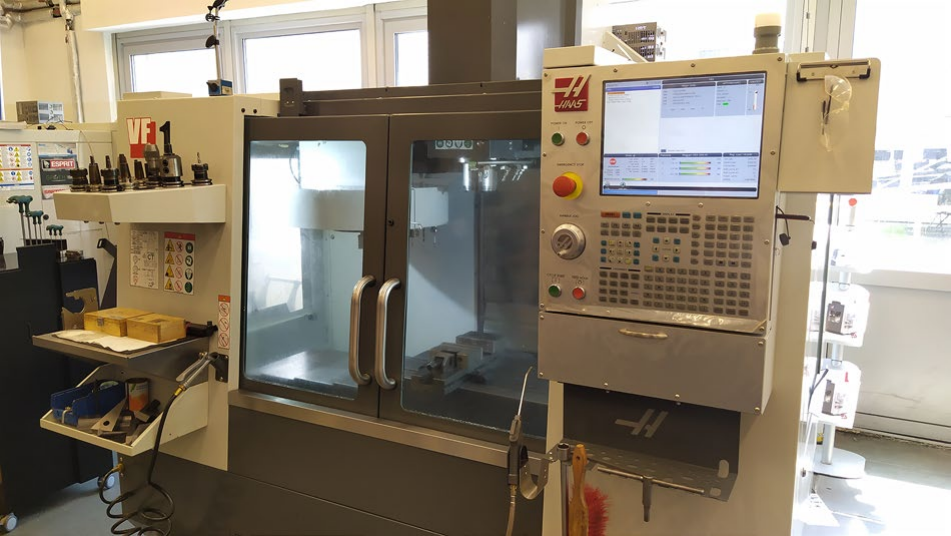
Haas VF-1milling machine with coordinate system designation.

The tests were carried out on a 3-axis Haas VF-1 milling machine (Fig. 1). The machine was fitted with 8 vibration sensors and 12 current transformers (designation in Table 1, respectively: A and C). All measurement tracks are characterised in Table 1. The sensor number corresponds to the channel number in the measurement system.

**Table 1:** Basic information about the location and type of sensors.

Sensor no.	Installation location	Installation direction/phase	Sensor type	Sensor model	Sensitivity [mV/g]
1	spindle	+Y	A	Dytran 3055D2	100.32
2	spindle	−Z	A	Dytran 3056D2T	101.29
3	spindle	−X	A	Dytran 3055D2	99.56
4	X axis	+Z	A	Dytran 3055D2	98.83
5	X axis	−X	A	Dytran 3055D2	100.99
6	Y axis	+Z	A	Dytran 3055D2	101.71
7	Y axis	+Y	A	Dytran 3055D2	99.49
8	Y axis	−X	A	Dytran 3055D2	99.17
9	spindle	L1	C	Wago 855-4001/0100-0001	n/a
10	spindle	L2	C		
11	spindle	L3	C		
12	X axis	L1	C		
13	X axis	L2	C		
14	X axis	L3	C		
15	Y axis	L1	C		
16	Y axis	L2	C		
17	Y axis	L3	C		
18	Z axis	L1	C		
19	Z axis	L2	C		
20	Z axis	L3	C		

**Fig. 2:**
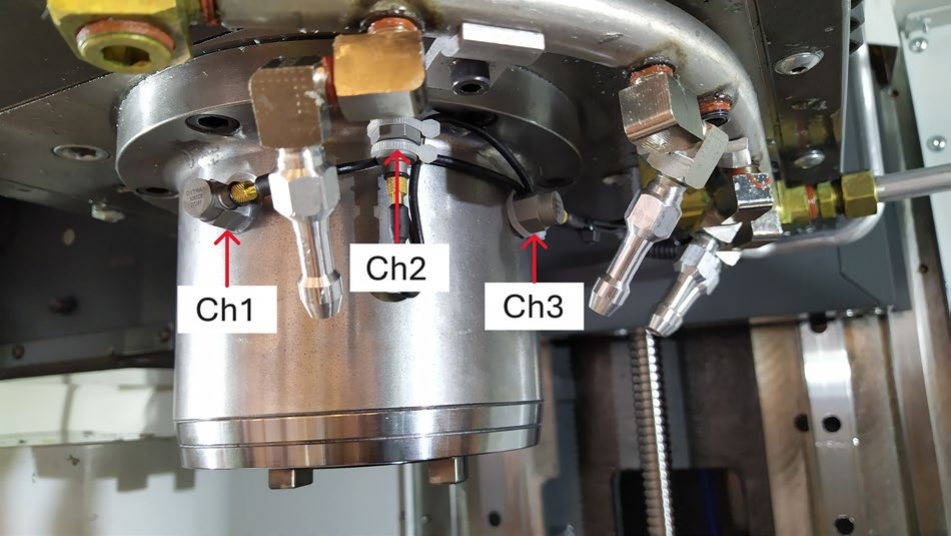
Location of accelerometers on the spindle of the Haas VF-1.

**Fig. 3:**
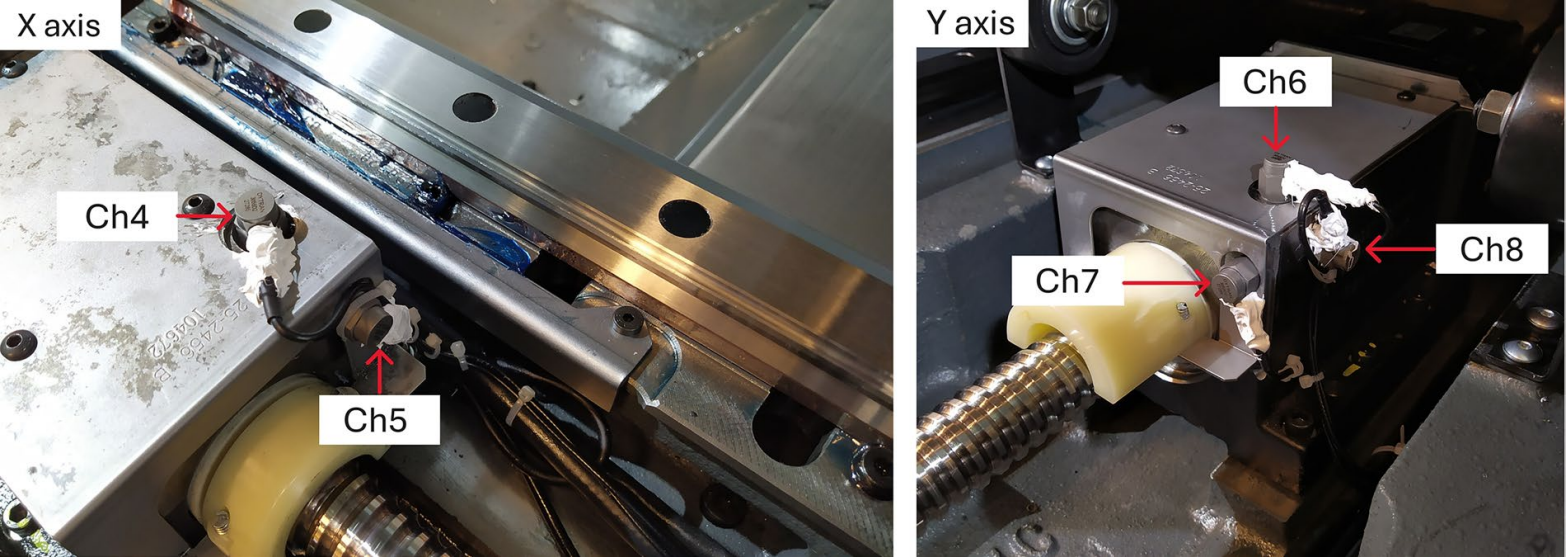
Location of accelerometers on the drive axes of the Haas VF-1.

The installation locations of the individual vibration sensors in Figs. 2 and 3 were shown. For the drive axis X, it was not possible to mount a sensor in the Y direction due to the lack of space.

**Fig. 4:**
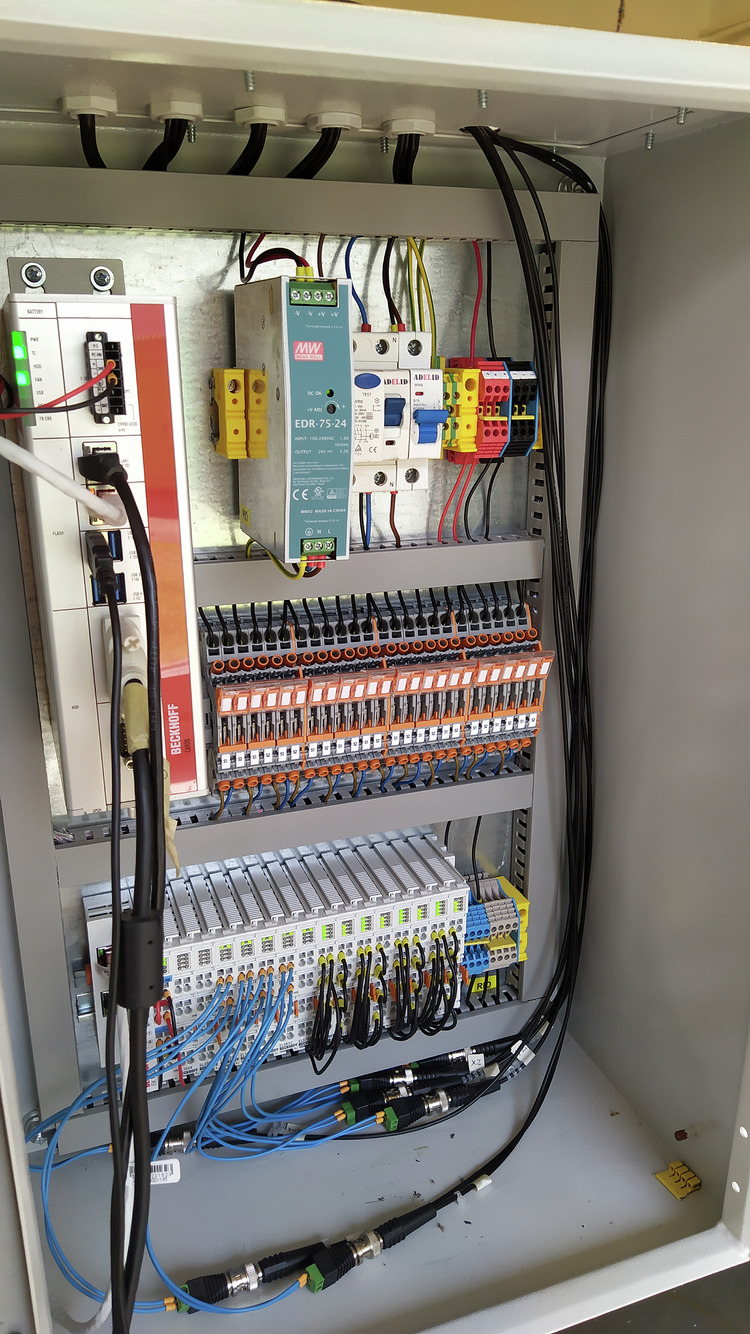
Data recorder.

The data were recorded using an industrial computer (Beckhoff C6920, Fig. 4) with measurement modules (EL3632, EL3413) connected via an EtherCAT network coupler (EK1100). The sampling frequency of vibration signals were 25 kHz and for current signals were 0.5 kHz

### Materials and sample preparation

The authors conducted a comprehensive analysis of the available publications on CNC machining, related to the performance of actual research experiments and measurement analysis^2,4–7,9,13,30^. It was decided to prepare a custom stand and analogous samples, with the material selected based on its common use in various industries, including aviation^31^ and automotive^32–34^. The material 42 CrMo4 was chosen, and the samples were prepared with IT12 tolerance and heat hardened to 38 ± 2 HRC. However, the samples exhibited a greater discrepancy in hardness than anticipated. The lowest measured hardness was 35.33 HRC, while the highest was 41.67 HRC. Consequently, hardness was measured at several points for each sample, and the results were averaged. The sample dimensions were 80 × 80 × 150 mm, with 20 mm depth excluded from cutting to allow for secure mounting in a vice.

The key to the proper conduct of the experiments was continuous milling, without the phase of retracting the tool from the material. To allow the tests to reflect real-life conditions as much as possible and be well adapted to industrial machining conditions, the course of the milling path was planned as a clockwise movement around the contour of the workpiece.

**Fig. 5:**
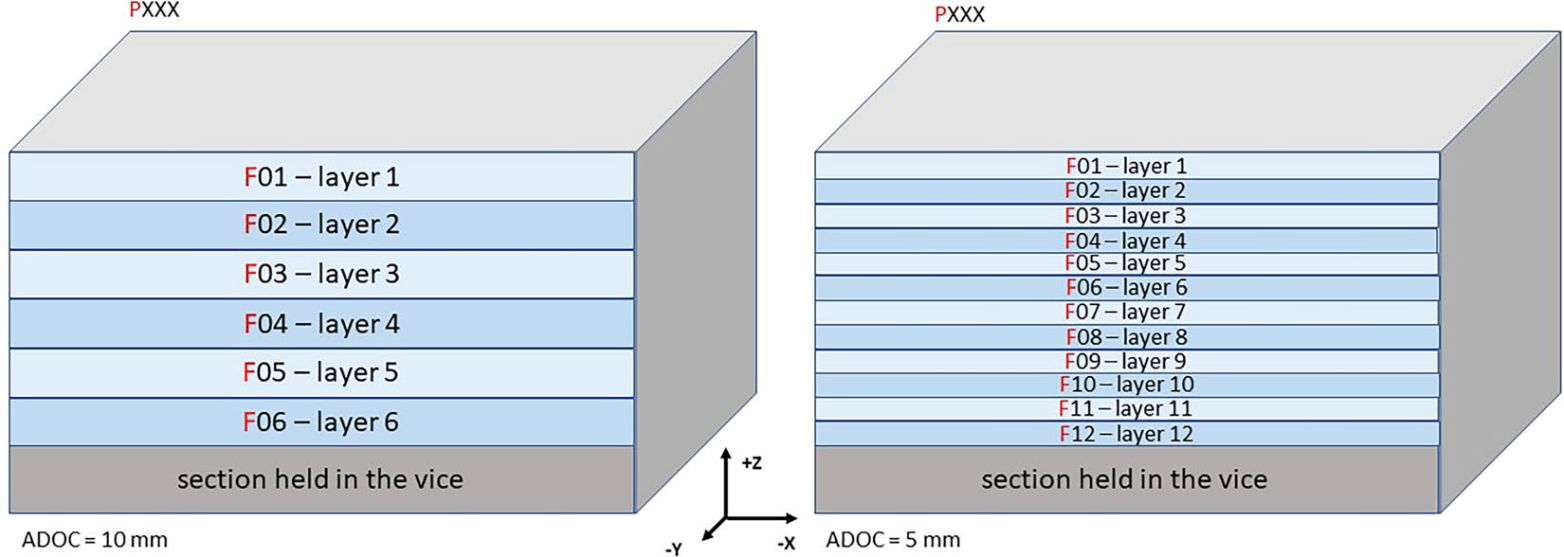
Division of the sample into layers depending on ADOC (coordinate system compatible with the machine system).

**Fig. 6:**
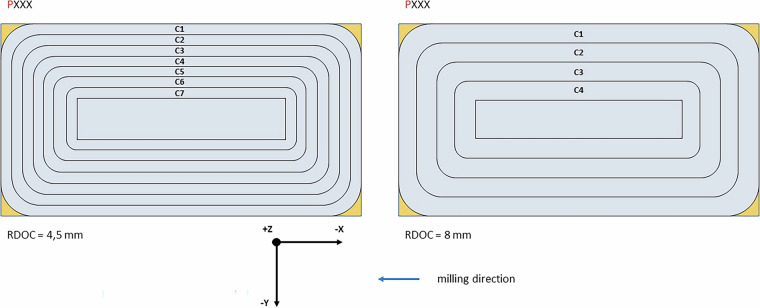
Division of the sample into cycles depending on RDOC (coordinate system compatible with the machine system).

**Fig. 7:**
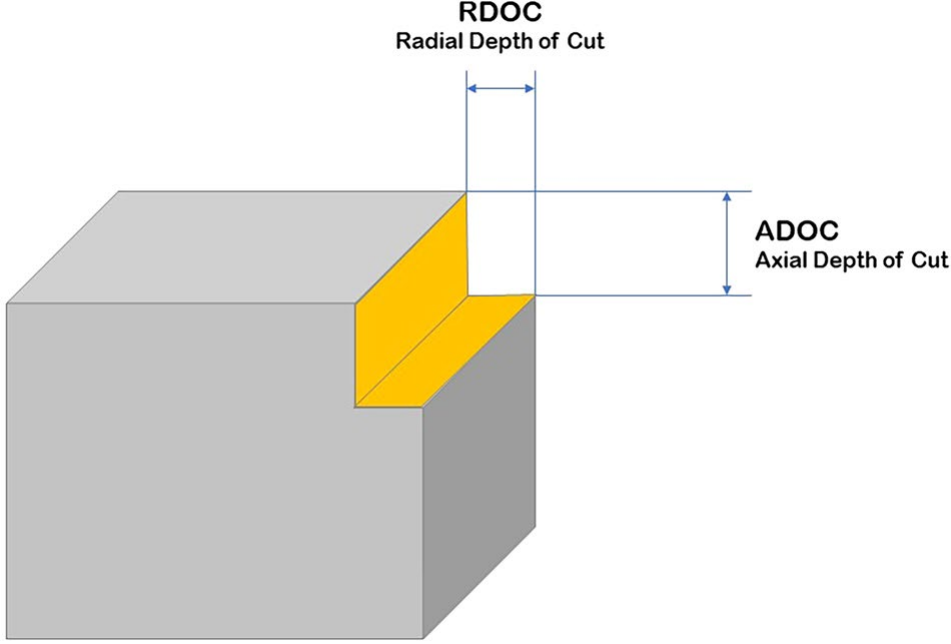
Schematic illustrating the ADOC and RDOC parameters.

The data collected during the experiments were named by sample number (P), layer number (F) (Fig. 5) and cycle number (C) (Fig. 6). The samples were single cuboid blocks of the material being processed. Each sample was divided into layers that corresponded to the height of the thickness of the material removed at a given stage of the experiments. The cycles, each of which a single path of the machining programme was run clockwise, were distinguished in the layers. The number of layers and cycles defined for a given sample depended on the adopted ADOC and RDOC values (Fig. 7). The most important parameters of the process and the samples are summarised in Table 2.

Experiments were conducted for various cases. Two milling cutters from different manufacturers were used, and milling was performed under both optimal and non-optimal conditions. This means that some experiments were performed according to the manufacturer’s recommendations, while others were not. Specifically, the RDOC parameter was set to 4.5 mm (not exceeding 45% of the tool diameter), representing optimal conditions, or to 8 mm, which significantly exceeded the manufacturer’s recommended operating conditions. Due to the different RDOC values, either 7 or 4 cycles (C) were performed on one layer (F) of the samples. The ADOC parameter was also varied, with values of 5 or 10 mm, which corresponded to the number of layers that could be milled: 6 or 12, respectively. Thus, a minimum of 24 cycles and a maximum of 84 cycles were performed on a single sample.

**Fig. 8:**
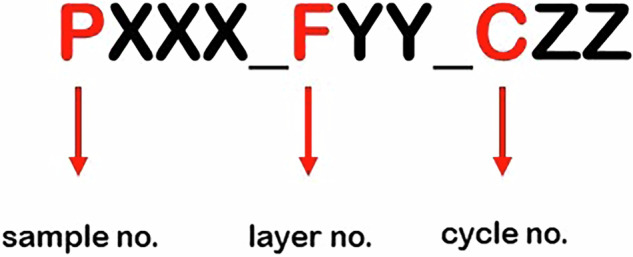
Numbering scheme for the milling experiment.

**Fig. 9:**
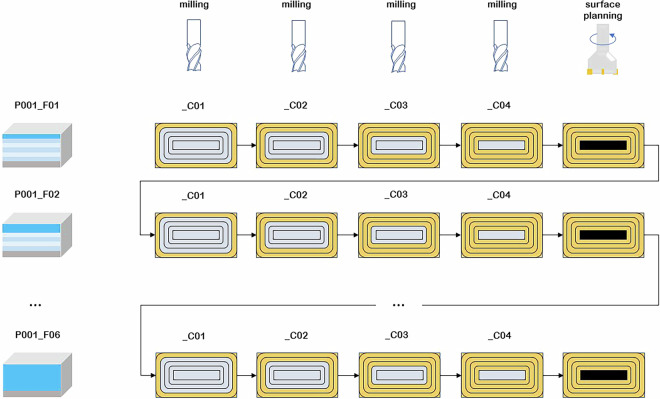
Experimental and data collection procedures.

Each single milling process is labelled according to the numbering scheme shown in Fig. 8. The sample was always milled starting from cycle no. 1. Sequential cycles performed are marked in orange in Fig. 8. After all the cycles on one layer had been performed, surface planing was carried out, which involved leveling the rest of the material, marked in black. Next, the tool base was lowered and data were registered again, starting from cycle no. 1 (Fig. 9).

**Fig. 10:**
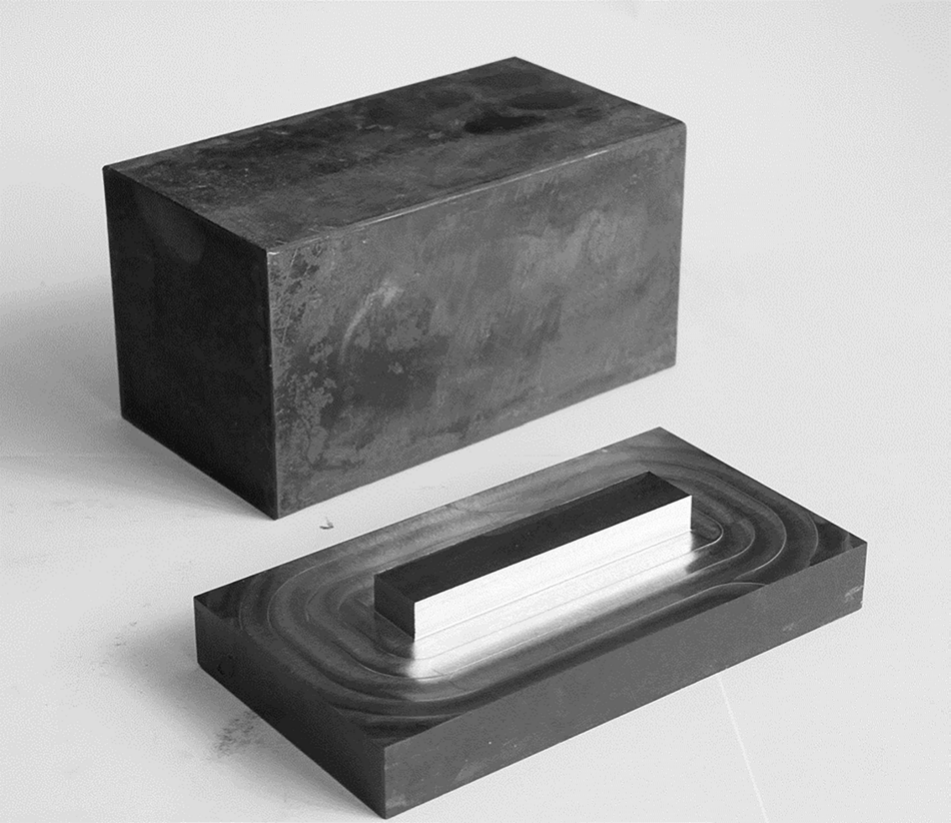
Sample before and after milling.

The basic statistics of the data collected in the experiments and the parameters of the process and samples are presented in Table 2. The actual appearance of the sample before and after treatment is shown in Fig. 10.

**Fig. 11:**
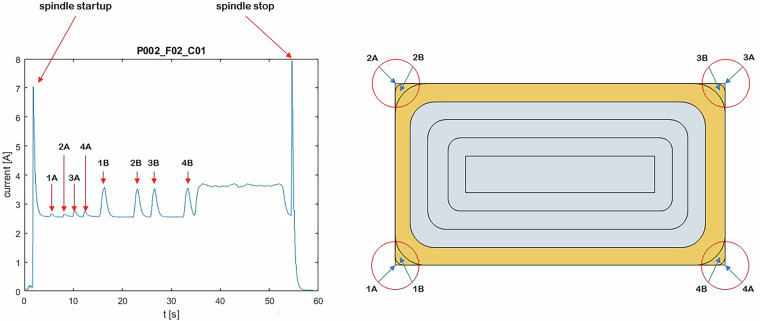
Interpretation of the spindle current signal (L1) for cycle 1.

**Fig. 12:**
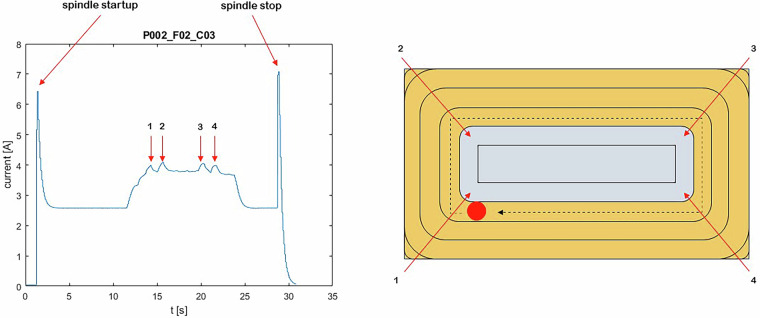
Interpretation of the spindle current signal (L1) for cycle 3.

**Fig. 13:**
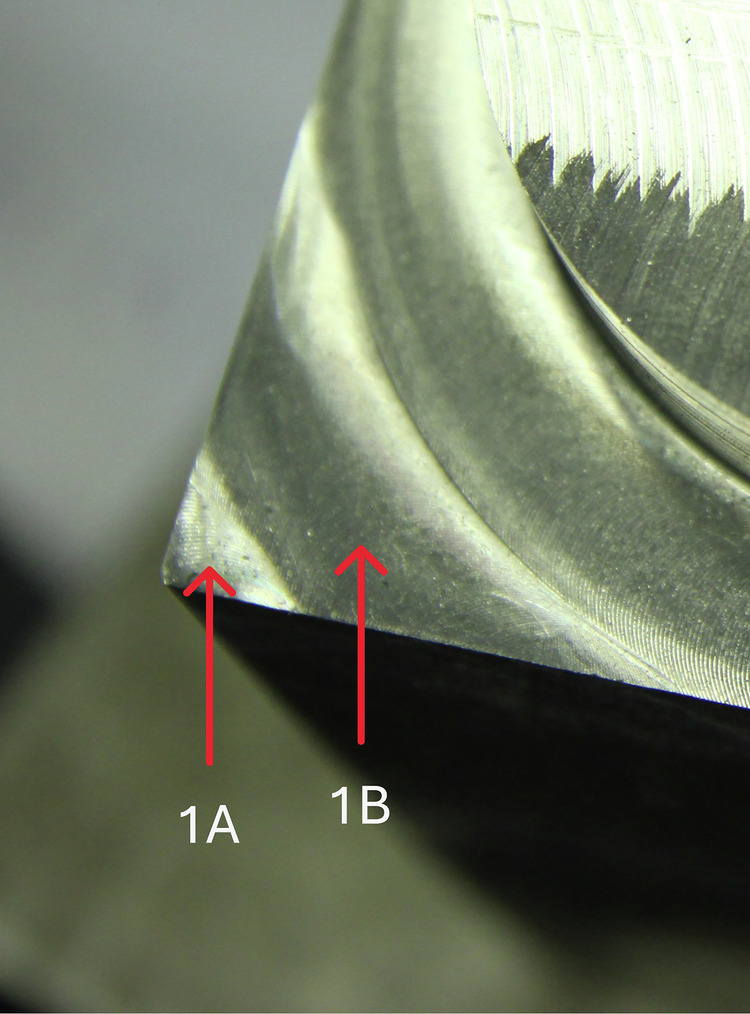
Location of sites marked as 1 A and 1B in Fig. 11. For the remaining corners of the workpiece, the markings are analogous.

For the experiments with an RDOC of 4.5 mm, the interpretation of the current signal (L1) from the spindle is shown in Figs. 11 and 12. A view of the actual location of 1 A, 1B, etc. The sample is provided in Fig. 13.

**Fig. 14:**
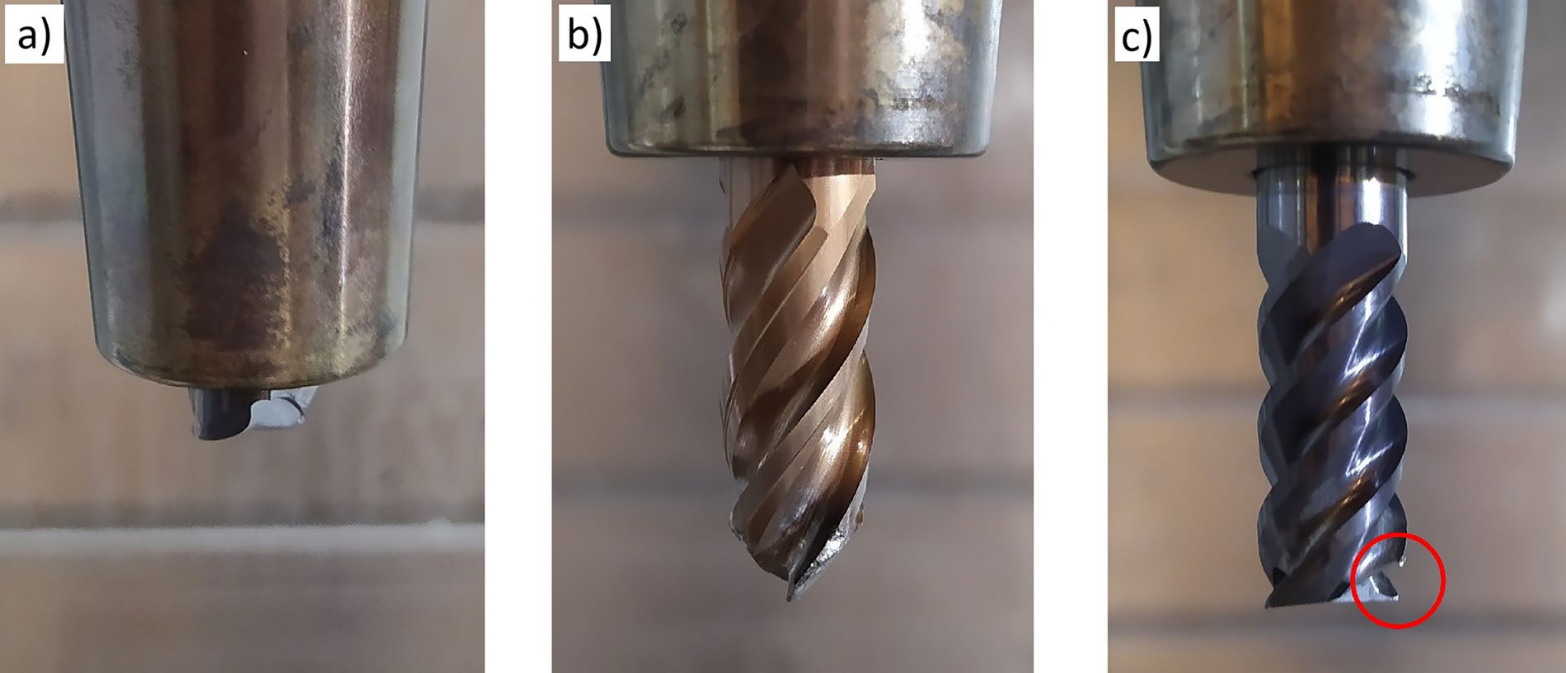
Different degrees of cutter damage.

**Fig. 15:**
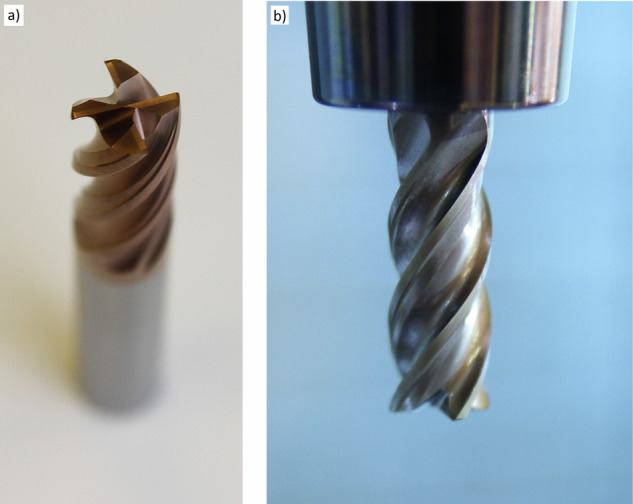
New cutters.

Cutter damage was characterized by complete breakage (Fig. 14a and b) or the breakage of one of the blades (Fig. 14c). For comparison, new cutters are shown in Fig. 15.

## Data Records

The dataset is available at Figshare (10.6084/m9.figshare.28589216)^35^.

### Raw data

**Fig. 16:**
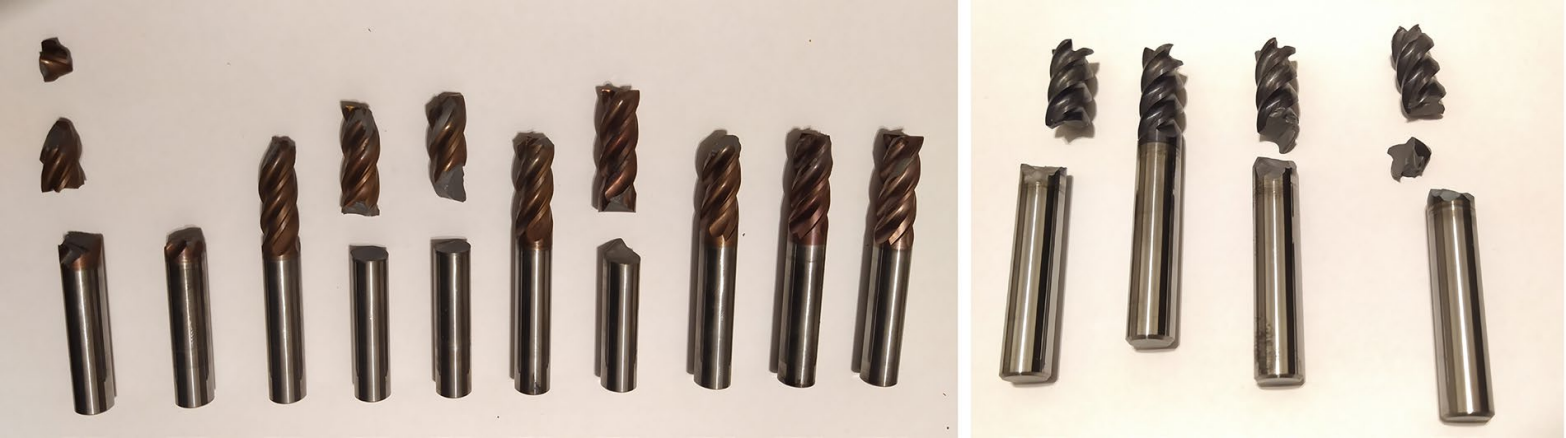
Damaged cutters (on the left MillingToolType – 1, on the right MillingToolType – 2).

The data covers 968 milling cycles during which 14 cutters were damaged (Fig. 16). One of them was damaged in the first cycle. This was considered a random event, which was treated as an outlier but it left in the data set (P114_F01_C1). Each file contains a set of raw data from 20 measurement channels and takes the form of a matrix [A x 22], where A is the variable number of rows depending on the file and 22 is the number of columns (20 columns with measurement data and 2 columns with timestamps). A fragment of a sample file was shown in the Table 2.

**Table 2:** Fragment of a sample raw data file.

*1*	*2*	*3*	*4*	*5*	*6*	*7*	*8*	*9*	*10*	*11*
TimestampsAcc	AccelerometerSpindleY	AccelerometerSpindleZ	AccelerometerSpindleX	AccelerometerXDrivingaxleZ	AccelerometerXDrivingaxleX	AccelerometerYDrivingaxleZ	AccelerometerYDrivingaxleY	AccelerometerYDrivingaxleX	TimestampsCurrent	CurrentSpindleL1
0	−442	−134	−190	−12	3	7	−21	23	0	129252
0.04	−429	−97	3	−12	1	1	−16	13	2	129252
0.08	−78	31	−285	−10	13	7	5	10	4	129252
0.12	14	−81	−240	−12	6	2	5	6	6	129252
***12***	***13***	***14***	***15***	***16***	***17***	***18***	***19***	***20***	***21***	***22***
CurrentSpindleL2	CurrentSpindleL3	CurrentDrivingaxleXL1	CurrentDrivingaxleXL2	CurrentDrivingaxleXL3	CurrentDrivingaxleYL1	CurrentDrivingaxleYL2	CurrentDrivingaxleYL3	CurrentDrivingaxleZL1	CurrentDrivingaxleZL2	CurrentDrivingaxleZL3
129960	130164	354	343	354	316	322	353	25018	25082	24587
129960	130164	354	343	354	316	322	353	25018	25082	24587
129960	130164	354	343	354	316	322	353	25018	25082	24587
129960	130164	354	343	354	316	322	353	25018	25082	24587

Column 1 contains timestamps for vibration signals (increasing every 0.04 ms, corresponding to a sampling frequency of 25 kHz). Columns 2–9 contain raw data from accelerometers, with columns 2–4 representing sensors mounted on the spindle and the rest on the drives of the worktable. Column 10 contains timestamps for current signals (increasing every 2 ms, corresponding to a sampling frequency of 0.5 kHz). Columns 11–13 represent the spindle (phases L1, L2, L3), and the subsequent columns represent the worktable, for each axis and each phase L1, L2, L3.

If necessary, the raw values can be converted to acceleration in units of [g] or [m/s²] for vibration signals (1) and to amperes [A] for current signals (2).

1 $${Acc}=\frac{{data}\ast 5[V]}{{2}^{15}\ast S}$$

where:

Acc – value in [g]

S – Sensitivity of sensors in [V/g] (use the value rounded to 0.1 V/g or the exact value from Table 1, given for each sensor separately, but the sensitivity in the table is given in [mV/g]).

2 $${current}={data}\ast 0.00002$$

where:

current – value in [A]

data – raw value

The authors recommend using the group cross-validation method, with each tool constituting a separate group (partition), as the best technique for training and testing algorithms. In this case, data should be divided into 13 partitions, with each partition containing data for only one tool. Algorithms should be trained on 12 partitions and tested on one partition. This approach aligns with industrial realities, where no data is available for new tools, and models should be trained on previously damaged tools and adapted to new, previously unknown data.

Used tools:

- Van Hoorn VHVTR41000701003050 (MillingToolType – 1)
- PARA Tooling RS4 10,0 × 70 (MillingToolType – 2)

### Metadata

The measurement database was supplemented with so-called metadata, which allowed to link signal data to the process parameters for which a given experiment was carried out. Some of them were constant for all experiments (*ToolRotation*, *FeedRate*, *ToolDiameter*). The test workpieces were characterised by a large discrepancy in hardness, and hence the numbers of the workpiece and tool as well as the average hardness are provided. If a given workpiece was milled with one tool and the tool did not break, another workpiece of similar hardness was selected. The metadata developed for the milling process experiments are listed in Table 3. The proposed column with the predicted values is marked in italics.

**Table 3:** A sample of a metadata file.

	ExperimentIndex	SampleIndex	ToolIndex	MillingToolType	ADOC [mm]	RDOC [mm]	HardnessMean [HRC]	CycleToFailure	*CycleToFailureNormalized*	ToolHolderLength [mm]	ToolRotation [rpm]	FeedRate [rpm]	ToolDiameter []
1	P002_F01_C1	2	2	1	5	8	36,67	49	1	80	3200	640	10
2	P002_F01_C2	2	2	1	5	8	36,67	48	0,979591837	80	3200	640	10
3	P002_F01_C3	2	2	1	5	8	36,67	47	0,959183673	80	3200	640	10
4	P002_F01_C4	2	2	1	5	8	36,67	46	0,93877551	80	3200	640	10
5	P002_F02_C1	2	2	1	5	8	36,67	45	0,918367347	80	3200	640	10
6	P002_F02_C2	2	2	1	5	8	36,67	44	0,897959184	80	3200	640	10
963	P119_F06_C3	119	105	2	5	4,5	38,5	5	0,040322581	80	3200	640	10
964	P119_F06_C4	119	105	2	5	4,5	38,5	4	0,032258065	80	3200	640	10
965	P119_F06_C5	119	105	2	5	4,5	38,5	3	0,024193548	80	3200	640	10
966	P119_F06_C6	119	105	2	5	4,5	38,5	2	0,016129032	80	3200	640	10
967	P119_F06_C7	119	105	2	5	4,5	38,5	1	0,008064516	80	3200	640	10
968	P119_F07_C1	119	105	2	5	4,5	38,5	0	0	80	3200	640	10

where:

CycleToFailure - number of cycles remaining until tool failure occurs,

CycleToFailureNormalized - number of cycles remaining until tool failure, normalized to the range [0, 1], where 0 denotes the cycle in which tool failure occurs, and 1 is the first cycle for the new tool).

A summary of the number of cycles completed by individual tools along with basic parameters is presented in Table 4. The HardnessMean column provides hardness ranges from the lowest to the highest - this applies to cases when the tool milled at least 1 sample.

**Table 4:** Number of cycles completed by individual tools along with basic parameters.

Tool No.	ToolIndex	MillingToolType	ADOC [mm]	RDOC [mm]	Hardness(range) [HRC]	CycleToFailure	ToolHolderLength [mm]
1	2	1	5	8	35,33–36,67	49	80
2	3	1	10	8	37,5-40	49	80
3	4	1	5	8	35,33	40	80
4	5	1	10	8	37,5–38,33	53	80
5	6	1	5	8	40	29	80
6	7	1	10	4,5	39,33–41,33	126	80
7	8	1	5	4,5	39–41,67	115	80
8	9	1	5	4,5	37,33–39,33	149	80
9	10	1	10	4,5	37,33	14	160
10	11	1	10	4,5	37,17	0	160
11	101	2	10	4,5	37,83–39,33	83	80
12	102	2	5	4,5	38,33	84	80
13	103	2	10	4,5	39,17–39,83	59	80
14	105	2	5	4,5	38,33–38,5	124	80

### Processed data set

Based on the collected measurement data (968 cycles), a set of 120 features in the time domain was determined for a single cycle, six features for each of the 20 channels:

- minimum,
- maximum,
- mean,
- standard deviation,
- skewness,
- kurtosis.

As a result of the feature extraction, a data matrix of 968 × 120 [rows × columns] was obtained, which was then combined with selected features of the metadata matrix with a final size of 968 × 10. Additionally, a new feature was introduced, which is the number of cycles performed by a given tool, a parameter that is always known by the machine operator. In this way, a final matrix of 968 × 131 was obtained. The last column (131) contained the target values – the number of cycles to failure of the tool normalized to the range[0–1] (*CycleToFailureNormalized*).

## Technical Validation

**Fig. 17:**
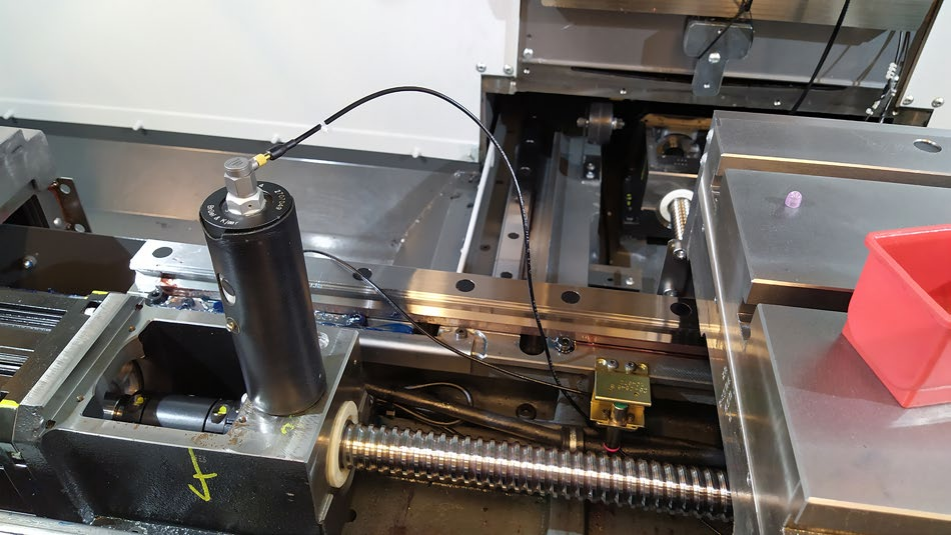
Accelerometer connected to Bruel&Kjaer 4296 vibration exciter before mount inside CNC.

Each time before mounting the vibration sensors, a procedure was carried out to test the accuracy of all measurement channels, including the measurement channels in the Beckhoff EL3632 modules and the sensors installed in the setups. The tests were carried out using a Bruel&Kjaer 4296 vibration exciter (Fig. 17), which generated a reference sinusoidal vibration signal with a specified frequency (159.2 Hz) and an RMS amplitude value of 10 m/s². This allows for confirmation that the vibration signals were recorded correctly. Additionally, the tool was mounted in thermal holders ensuring repeatability of assembly, and consequently, the comparability of measurements.

The hardness of the samples was measured 3 to 5 times to ensure repeatability of the measurement. The hardness noted in the metadata is the arithmetic mean of all measurements for a given sample.

## Usage Notes

The data are also available at Kaggle,

https://www.kaggle.com/datasets/grzegorzpiecuch/cnc-milling.

## Data Availability

No custom code is needed to access the data.
